# Delayed infection, late tonsillectomy or adenoidectomy and adult leukaemia: a case–control study

**DOI:** 10.1038/sj.bjc.6600689

**Published:** 2003-01-28

**Authors:** P Vineis, L Miligi, P Crosignani, L Davico, A Fontana, G Masala, O Nanni, V Ramazzotti, S Rodella, E Stagnaro, R Tumino, C Viganò, C Vindigni, A S Costantini

**Affiliations:** 1 Servizio di Epidemiologia dei Tumori, Ospedale S. Giovanni Battista e Università di Torino, via Santena 7, I-10123 Torino, Italy; 2 Centro per lo Studio e la Prevenzione Oncologica, USL 10 Firenze, Italy; 3 National Cancer Institute, Milano, Italy; 4 Local Health Unit, Novara, Italy; 5 Istituto Oncologico Romagnolo, Forlì Italy; 6 Cancer Registry, Latina, Italy; 7 Local Health Unit, Verona, Italy; 8 National Cancer Institute, Genova, Italy; 9 Cancer Registry, Ragusa, Italy; 10 Department of Pathology, University of Siena, Italy

## Abstract

In a population-based case–control study among adults in Italy, of 261 lymphoid and 313 myeloid leukaemias and 1718 controls, a later age at adenoidectomy and tonsillectomy (after age 10 years) increased considerably the risk of lymphocytic (but not myeloid) leukaemia (odds ratio 4.2, 95% confidence interval 1.1–16.2). We propose that late infection is a proliferative stimulus for B-cells.

Some adult diseases have been attributed to late exposure to common infectious agents–poliomyelitis (Nielsen *et al*, 2002), Hodgkin's disease (Gutensohn and Cole, 1981) and multiple sclerosis (Lindberg *et al*, 1991)–but the evidence is not definitive. Perrillat *et al*,(2002) found an inverse association between day-care attendance, repeated early common infections or surgical procedures for ear–nose–throat infections, and childhood leukaemia, with odds ratios (ORs) in the order of 0.5–0.6.

We conducted a population-based case–control study on leukaemias in adults in Italy. We examined the role played by two surgical operations, adenoidectomy and tonsillectomy, in modifying the risk of adult leukaemia. A late age at adenoidectomy and tonsillectomy has been proposed as a proxy for delayed infection with Epstein–Barr virus (EBv) (Perrillat *et al*, 2002). Our hypothesis is that late EBV infection or other exposures to infectious agents are a proliferative stimulus for B-cells, which causes symptomatic mononucleosis in normal conditions, but may be able to confer proliferative advantage to mutated cells.

## METHODS

The areas included in the study are 11 Italian areas with different demographic and socioeconomic characteristics (town of Torino, provinces of Varese, Novara, Vercelli, Alessandria, Imperia, Ragusa, Siena, Forlì, Verona and Firenze; total population 7 million). In each centre, all the subjects suspected of having leukaemia, lymphoma or multiple myeloma were identified through periodical surveys in the hospital departments where such cases are diagnosed. Only newly diagnosed cases occurring in the study period (1990–1993) were included (both sexes, aged 20–74 years, residents of the areas under study). In each hospital department a reference physician was identified and cooperated in case finding. A timely recruitment was necessary in order to keep the proportion of cases who were alive at interview as high as possible. All suspect cases were interviewed; the case status was further evaluated after interview, with direct access to the clinical record and/or the histologic diagnosis. Expected numbers of cases for each of the diseases included in the study were computed, based on Cancer Registries (data available on request). The observed/expected ratio was generally close to 1 or slightly lower than 1. Observed/expected ratios lower than 1 can be explained by the rather stringent criteria for inclusion into our study (for example, Cancer Registries include a variable proportion of DCO (death certificate only) cases).

The control group was a random sample of the population resident in each of the areas, aged 20–74 years. The sample was stratified according to 5-year age groups and sex, and its size was equal to the number of cases in the largest diagnostic group (NHL+CLL). Procedures for control sampling were based on computerised and regularly updated demographic files or through the files of the National Health Service, both of which are regularly updated.

Information about the known or suspect risk factors was collected through person-to-person interviews. Interviews were carried out preferentially at the subject's home. The same procedures were followed for cases and controls. The only exception was represented by interviews to cases affected by acute leukaemia and the seriously ill, which were mostly carried out in the hospital. Subjects were encouraged to participate, by contacts with the general practitioners, and were successful in maintaining a high response rate. The interview was face-to-face and lasted approximately 1 h, as described elsewhere in more detail (Vineis *et al*, 2000a, 2000b), and covered a medical history of the following diseases: measles, rubeola, chickenpox, pertussis, herpes zoster, herpes labialis, herpes genitalis, mumps, infectious hepatitis, infectious mononucleosis, tuberculosis, malaria, other infectious diseases (specified by the subject), hay fever, allergic asthma, other (specified) allergies, peptic ulcer, ulcerative colitis, gluten intolerance, Crohn's disease, other (specified) digestive diseases, diabetes and other (specified) metabolic diseases, rheumatoid arthritis, lupus erythematosus, periarteritis nodosa, sclerodermia, other (specified) osteomuscular diseases, urticaria, eczema, psoriasis, other skin allergies, and other (specified) relevant diseases. Only diseases that had been formally diagnosed by a physician were considered. For each disease, the date of occurrence was determined, except for the ‘other, specified’ category. The overall refusal rate was 10% among cases and 19% among controls.

We computed age- and gender-adjusted ORs (Mantel-Haenszel) and their 95% CIs (Breslow and Day, 1980). We also fitted logistic regression models including age (continuous variable), gender and educational level.

## RESULTS AND DISCUSSION

Overall, we interviewed 261 patients with lymphocytic leukaemia, 313 with myeloid leukaemia and 1718 controls. Table 1

**Table 1:** Lymphyocytic and myeloid leukaemias: age at surgical interventions for adenoidectomy or tonsillectomy

	Lymphocytic	OR (95% CI)	Myeloid	OR (95% CI)
*Adenoidectomy*				
No	241/1524	1.0 (ref)	276/1524	1.0 (ref)
Yes	20/194	0.8 (0.5–1.3)	37/194	1.2 (0.8–1.7)
				
*Age at intervention (y)*				
<10	4/103	1.0 (ref)	17/103	1.0
10–20	10/52	4.2 (1.1–16.2)	9/52	1.0 (0.4–2.2)
20+	3/16	4.1 (0.2–4.1)	2/16	0.92 (0.2–4.4)
Age unknown	3/23		9/23	
				
*Tonsillectomy*				
No	206/1270	1.0 (ref)	244/1270	1.0 (ref)
Yes	55/449	0.9 (0.7–1.3)	69/449	0.9 (0.6–1.1)
				
*Age at intervention (y)*				
<10	15/203	1.0 (ref)	28/203	1.0 (ref.)
10–20	18/118	1.8 (0.8–4.1)	17/118	1.1 (0.6–2.0)
20+	15/114	1.5 (0.5–4.0)	16/114	1.0 (0.6–2.3)
Age unknown	7/14		8/14	

Cases/controls. ORs are age- and gender-adjusted; 95% CI.

shows the distribution of adult lymphocytic and myeloid leukaemias by adenoidectomy and tonsillectomy, and the age at which such operations were performed. The operations themselves were not associated with any risk modification. However, a later age at adenoidectomy and tonsillectomy (after age 10 years) increased considerably the risk of lymphocytic (but not myeloid) leukaemia. The fact that the increase in risk is limited to lymphocytic leukaemia enhances the biological plausibility of the association.

In a previous paper on non-Hodgkin's lymphomas (NHL), we found that the age of occurrence of bacterial and viral diseases was significantly higher among NHL patients than in the controls. The association between later age at first bacterial or viral disease was limited to small families (OR=1.95, 95% CI 1.26–3.00, for age 4–8 years at first infection; OR=1.91, 1.19–3.06, for age 9+years, compared with less than 4 years) (Vineis *et al*, 2000b). The association was more obvious for bacterial diseases (possibly because of a lower degree of misclassification). We repeated the same analyses for leukaemia, although limited by smaller numbers; ORs are presented in Table 2

**Table 2:** Lymphocytic leukaemias: age at first infection (see list in the text), by number of siblings

Age at first infection (y)	ca/co	OR (CI)
*Siblings 0 or 1*		
<4	8/94	1.0 (ref.)
4–8	28/223	1.5 (0.6–3.4)
>8	24/145	1.8 (0.7–4.4)
		
*Siblings 2 or more*		
<4	15/94	1.0 (ref.)
4–8	47/473	0.6 (0.3–1.2)
>8	68/360	1.0 (0.6–1.9)

Ca/co=cases/controls. Odds ratios(ORs) are age- and gender-adjusted; 95% CI=confidence interval.

. In spite of statistical instability, there is the suggestion of a higher risk of lymphocytic leukaemia in smaller families and with older age at first infection. The same pattern was not apparent for myeloid leukaemias (data not shown). While data on age at first infection are likely to be affected by recall bias, surgical operations such as tonsillectomy or adenoidectomy should be recalled more faithfully.

In one previous study, tonsillectomy or appendectomy was found to increase the risk of childhood leukaemia, but the age at surgical intervention was not investigated (Schuz *et al*, 1999). A late age at adenoidectomy and tonsillectomy has been proposed as a proxy for delayed infection with EBV or other viruses (Perrillat *et al*, 2002). Our hypothesis is that late EBV infection is a proliferative stimulus for B-cells. Such an event causes symptomatic mononucleosis in normal conditions, but is able to confer proliferative advantage to mutated cells. An interesting model is represented by paroxysmal nocturnal haemoglobinuria (PNH), characterised by a somatic mutation of the PIG-A gene in haematopoietic cells (Rosse, 2001). The current hypothesis explaining the disorder suggests that there are two components: (a) haematopoietic stem cells with the characteristic defect are present in the marrow of many, if not all, normal individuals in very small numbers; (b) some aplastogenic influence suppresses the normal stem cells, but does not suppress the defective stem cells, thus allowing the proportion of these cells to increase. Clearly, the PNH model applies only indirectly to leukaemias because there is no reason to think that late infection is aplastogenic. Rather, mutations or chromosome damage would be necessary to induce leukaemia, but a proliferative stimulus (infection) causing a selection of the mutated clones would also intervene.

## References

[bib1] Breslow N, Day N(1980) *Statistical methods in cancer research*. The Analysis of Case–Control Studies, Vol I. IARC Science Publication No. 32. Lyon: International Agency for Research on Cancer 7216345

[bib2] Gutensohn N, Cole P(1981) Childhood social environment and Hodgkin's disease. N Engl J Med 304: 135–140 6255329 10.1056/NEJM198101153040302

[bib3] Lindberg C, Andersen O, Vahlne A et al, (1991) Epidemiological investigation of the association between infectious mononucleosis and multiple sclerosis. Neuroepidemiology 10: 62–65 2062419 10.1159/000110248

[bib4] Nielsen NM, Aaby P, Wohlfahrt J, Molbak K, Melbye M(2002) The polio model. Does it apply to polio? Int J Epidemiol 31: 181–186 11914318 10.1093/ije/31.1.181

[bib5] Perrillat F, Clavel J, Auclerc MF, Baruchel A, Leverger G, Nelken B, Philippe N, Schaison G, Sommelet D, Vilmer E, Hemon D(2002) Day-care, early common infections and childhood acute leukaemia: a multicentre French case–control study. Br J Cancer 86: 1064–1069 11953850 10.1038/sj.bjc.6600091 PMC2364194

[bib6] Rosse WF(2001) New insights into paroxysmal nocturnal hemoglobinuria. Curr Opin Hematol 8: 61–67 11224678 10.1097/00062752-200103000-00001

[bib7] Schuz J, Kaletsch U, Meinert R, Kaatsch P, Michaelis J(1999) Association of childhood leukaemia with factors related to the immune system. Br J Cancer 80: 585–590 10408870 10.1038/sj.bjc.6690395 PMC2362320

[bib8] Vineis P, Crosignani P, Sacerdote C, Fontana A, Masala G, Miligi L, Nanni O, Ramazzotti V, Rodella S, Stagnaro E, Tumino R, Vigano C, Vindigni C, Costantini AS(2000a) Haematopoietic cancer and medical history: a multicentre case control study. J Epidemiol Community Health 54: 431–436 10818118 10.1136/jech.54.6.431 PMC1731690

[bib9] Vineis P, Miligi L, Crosignani P, Fontana A, Masala G, Nanni O, Ramazzotti V, Rodella S, Stagnaro E, Tumino R, Viganò C, Vindigni C, Seniori Costantini A(2000b) Delayed infection, family size and malignant lymphoma. J Epidemiol Commun Health 54: 907–911 10.1136/jech.54.12.907 PMC1731607 11076986

